# Patient Impression of Improvement 1 year After Sacrospinous Hysteropexy Versus Vaginal Hysterectomy in Women with Pelvic Organ Prolapse Stage 2 or Higher

**DOI:** 10.1007/s00192-024-05750-2

**Published:** 2024-02-28

**Authors:** Lisa M. Stoter, Kim J. B. Notten, Marieke Claas, Deodata Tijsseling, Maud Ruefli, Femke van den Tillaart, Sander M. J. van Kuijk, Alfredo L. Milani, Kristin B. Kluivers

**Affiliations:** 1 Department of Gynecology, Radboud University Medical Centre, Geert Grooteplein Zuid 10, 6525 GA Nijmegen, The Netherlands; 2 Department of Gynecology, Reinier de Graaf Gasthuis, Reinier de Graafweg 5, 2625 AD Delft, The Netherlands; 3 Bergman Clinics, Hilversum, Marathon 1, 1213 PA Hilversum, The Netherlands; 4 Department of Clinical Epidemiology and Medical Technology Assessment, Maastricht University Medical Centre, P. Debyelaan 25, 6229 HX Maastricht, The Netherlands

**Keywords:** Apical prolapse, Patient’s impression of improvement, POP-Q, Prolapse recurrence, Sacrospinous hysteropexy, Vaginal hysterectomy

## Abstract

**Introduction and hypothesis:**

Patient-reported outcomes are relevant outcomes in studies on pelvic organ prolapse (POP) surgery, as anatomical recurrence alone does not have a significant correlation with perceived improvement. In the present study, the patient’s impression of improvement after 1 year is studied after vaginal hysterectomy (VH) versus sacrospinous hysteropexy (SSH) in large cohorts from daily clinical practice. We hypothesize that there is no difference between the groups.

**Methods:**

This is a secondary analysis on prospectively collected data in a multicenter cohort of patients who underwent VH or SSH for symptomatic POP. All patients had a POP-Q stage ≥ 2 in at least one compartment at baseline and were treated with VH or SSH between 2002 and 2019. The primary outcome was the patient-reported score on the patient global impression of improvement index (PGI-I) 1 year after surgery. The secondary outcome was a composite outcome of surgical success, defined as the absence of recurrent POP beyond the hymen with bothersome bulge symptoms and/or repeat surgery.

**Results:**

A total of 378 women (196 VH and 182 SSH) were included. The median score on the PGI-I did not differ between VH and SSH. At 1 year post-operatively, 77 women after VH (73%) and 77 women after SSH (75%) considered their condition (very) much improved (*p* = 0.86). There was no difference in composite outcome of surgical success (126 out of 137 women [92%] after VH, 118 out of 125 women [94%] after SSH; *p* = 0.44).

**Conclusions:**

Our study shows that there was no difference in the type of surgery, VH or SSH, with regard to the patient’s impression of improvement 1 year postoperatively in a large cohort from daily clinical practice.

## Introduction

Female pelvic organ prolapse (POP) is a major health problem that affects over 40% of women above the age of 45 [1, 2]. Owing to an aging population and an increase in obesity, incidences of POP are rising globally [3]. When POP is symptomatic, it has a detrimental effect on the quality of life [4–6]. With an estimated lifetime surgery risk of 11 to 19%, many women will face the choice of selecting a surgical method that meets their expectations [4]. Vaginal hysterectomy (VH) has been the most common surgical treatment [5]. However, uterus-preserving techniques such as sacrospinous hysteropexy (SSH) and the Manchester procedure are gaining popularity because these techniques have been considered to be less invasive [7, 8]. Nonetheless, studies comparing VH and SSH regarding the recurrence of apical prolapse report conflicting results and use a variety of definitions of surgical success. A randomized controlled trial (RCT; *n* = 66) performed by Dietz et al. [9] concluded that SSH leads to a higher recurrence rate of apical prolapse than VH, 12 months after surgery. In contrast, in the multicenter RCT SAVE-U trial, Detollenaere et al. [7] and Schulten et al. [10] found that SSH was non-inferior to VH at 12 months after surgery, and superior at 5 years after surgery. The complication rates in these studies were comparable [7, 9, 10]. Supportive evidence from daily clinical practice, as from large cohort studies over many years, can provide insights into the differences between available surgical techniques.

Patient-reported outcomes, including the patient’s impression of improvement, are generally seen as the relevant outcome measures in POP. Barber et al. [11] found that subjective cure was associated with significant improvements in the patient’s assessment of treatment success and overall improvement, more so than any other definition considered. Detollenaere et al. [7] focused on patient satisfaction as a secondary outcome in the SAVE-U study. A significant improvement in the symptoms and patient satisfaction in the VH group and the SSH group was found after 12 months, without a difference between the groups. Additional research is needed to validate findings on patient’s impression of improvement, and the present study fills this gap.

The aim of this present project is to create evidence to help patients and their gynecologists to make well-considered and shared decisions with regard to a surgical procedure. The objective is to examine the difference in patient’s impression of improvement and composite outcome of surgical success between VH and SSH after 1 year in a large cohort study with prospectively collected data over a 15-year period.

## Materials and Methods

### Study Design and Patient Recruitment

This study is a secondary analysis on a prospectively collected multicenter cohort of female patients with a symptomatic POP who underwent VH or SSH. Only patients with a follow-up consultation at 1 year were included. All patients had a POP-Q stage ≥ 2 in at least one compartment at baseline, and were treated with VH for POP or SSH between October 2002 and April 2019 at the Radboud University Medical Centre or Reinier de Graaf Gasthuis, Delft. Patients with a history of vaginal or abdominal hysterectomy were excluded. Patients had been counseled for a vaginal POP operation including apical support. Treatment plans, including eventual concomitant vaginal repair, were individualized based on patient characteristics and preferences.

Prior to the surgical intervention, a gynecological examination was performed in all patients. This included staging of the POP using POP-Q, with patients in a 45° upright sitting position and measurements with and without Valsalva maneuver [12]. An endovaginal ultrasound was performed to rule out malignancy or other pathological conditions. Patients filled in the Dutch version of the validated pelvic floor specific questionnaires: Urogenital Distress Inventory (UDI), Incontinence Impact Questionnaire (IIQ), Defecation Distress Inventory (DDI), and the Pelvic Organ Prolapse/Urinary Incontinence Sexual Questionnaire (PISQ-12) [13, 14]. These questionnaires assess symptoms and bother of POP, urinary and defecatory problems, sexual dysfunction, and pain. The UDI, DDI, and IIQ are each subdivided into subscales ranging from 0 to 100, where higher scores indicate worse symptoms and more bother. Satisfaction with sexual function was measured on a four-point scale, where 1 = not satisfied, 2 = little satisfaction, 3 = satisfied, 4 = very satisfied.

After surgical intervention, participants had a regular follow-up consultation at 6 weeks and 1 year postoperatively to assess for recurrence and complications. At 1-year follow-up, POP-Q scores were repeated to objectify eventual anatomical recurrence of the POP. The same questionnaires were completed, now including the Patient Global Impression of Improvement (PGI-I) questionnaire [15]. The questionnaire serves as a patient-centered assessment tool evaluating improvement after treatment as a measure of effectiveness. The score is on a seven-point Likert scale, where 1 = very much better, 2 = much better, 3 = a little better, 4 = no change, 5 = a little worse, 6 = much worse, and 7 = very much worse.

### Data Collection

Recording data of all performed POP surgeries has been part of the hospital protocol since 2002. Three different data-entry systems were used to store these data between 2002 and 2019. Unfortunately, owing to a discontinued national data entry system, data from March 2014 to April 2016 could not be added to the database. We estimate that this resulted in a loss of less than 40 patients (approximately 10%) from our study cohort. Patient characteristics and medical details were collected during surgery and follow-up and were completed with information from the medical files. Data regarding the surgical procedures, including operative time, blood loss, length of hospital stay, and surgical complications were extracted. Complications were classified using the Clavien–Dindo classification [16].

### Surgical Procedures

#### Vaginal Hysterectomy

Vaginal hysterectomy is a surgical POP procedure in which the uterus is removed and the vaginal vault supported. After vaginal access was obtained, the cervix was separated from the vaginal wall by a circular incision. The anterior and posterior peritoneum were opened, and surrounding tissues including ligaments and vessels were clamped, detached from the uterus and sutured. The uterus was removed vaginally. A circular suture was placed intra-peritoneally and running circularly through the peritoneum, the uterosacral ligaments, and the full thickness of the vaginal epithelium of the posterior fornix (modified McCall culdoplasty). When closing the vaginal vault, the detached uterosacral ligaments were attached to the vaginal vault with two additional sutures. The combination of these sutures through the uterosacral ligaments restored normal support of the apical compartment [17].

#### Sacrospinous Hysteropexy

The procedure was performed using a transvaginal approach. The posterior vaginal wall was opened, and the right sacrospinous ligament was located through palpation. Two permanent sutures were placed with a disposable suture device (minimal access). These sutures were placed through the sacrospinous ligament, 2 cm from the ischial spine, and the posterior side of the cervix. The uterus remained in vivo. The posterior vaginal wall was then closed using absorbable sutures. The operation is regularly performed in combination with anterior colporrhaphy.

All surgical interventions were performed by a gynecologist, a fellow, or a resident under direct supervision based on formal competence. If necessary, concomitant anterior or posterior colporrhaphy or incontinence surgery was performed simultaneously. According to standard hospital protocol, all patients received perioperative antibiotics, thrombosis prophylaxis, a surgical vaginal gauze, and a bladder catheter. During the study, the catheterization period and hospitalization decreased by protocol from 3 to 1 night. Postoperatively, analgesics were administered, and patients were advised to refrain from heavy lifting and strenuous activities until 6 weeks postoperatively.

### Outcomes

The primary outcome was the score on the PGI-I. The median scores are presented per treatment group as well as the percentage of women reporting their improvement (very) much better (PGI-I success). The secondary outcome was a composite outcome of surgical success, defined as the absence of recurrent POP beyond the hymen with bothersome bulge symptoms and/or repeat surgery at the 1-year follow-up. This definition of POP assessment after treatment was recommended by Barber et al. [11] and was used in other large RCTs such as Detollenaere et al. [7] and Enklaar et al. [18]. The three components of the composite outcome are reported separately. The presence of bothersome bulge symptoms before and after surgery was defined as a positive answer to any of the following two questions from the UDI: “Do you experience a sensation of bulging or protrusion from the vagina?” and “Do you have a bulge or something falling out that you can see in the vagina?” in combination with a response “somewhat bothered” to “very much bothered” to the question “how much does this bother you?.” The repeat surgeries, by treated or nontreated compartment, are reported.

Further secondary outcomes were POP recurrence by compartment as measured by POP-Q, operating time, blood loss and (post-)surgical complications, and the other scores on the patient-reported questionnaires.

### Statistical Analysis

We made use of descriptive statistics for continuous and categorical data. To calculate differences between the two surgical procedures we used a Chi-squared test for nominal and ordinal variables, and the unpaired Student’s *t* test for continuous variables. Preoperative and postoperative domain scores on the questionnaire scores were analyzed using a Mann–Whitney *U* test and Wilcoxon signed-rank test. Logistic regression analysis was performed to correct for age, body mass index, and the preoperative presence of apical prolapse. Unadjusted and adjusted odds ratio and the 95% confidence interval (CI) were calculated. Missing POP-Q values and baseline characteristics were imputed using multiple imputation with predictive mean matching. Data and statistical analyses were performed using IBM SPSS software (version 26.0.0.0). A *p* value < 0.05 was considered statistically significant.

### Ethics

This study was conducted using pseudonymized data. Permission to conduct this ongoing outcome registration was granted by the Research Ethics Committee of the Radboud University Medical Centre Nijmegen and was deemed exempt from CME/IRB approval.

## Results

**Fig. 1:**
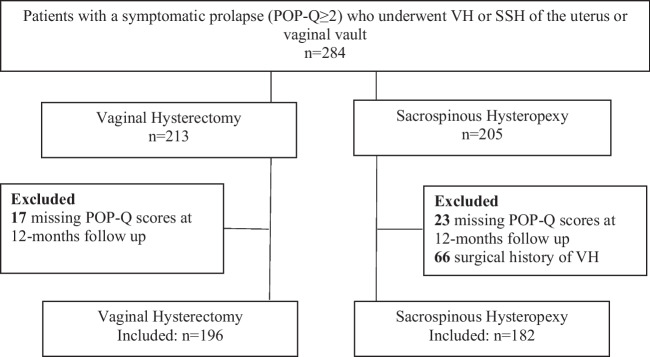
Flow diagram showing the patient recruitment process. *VH* vaginal hysterectomy, *SSH* sacrospinous hysteropexy, *POP-Q* pelvic organ prolapse quantification

Four hundred and eighty-four women (*n* = 484) underwent either VH or SSH for the indication of POP during the inclusion periods (2002–2014 and 2016–2019). Sixty-six patients underwent sacrospinous hysteropexy of the vaginal vault and were excluded because of a surgical history of VH. Three hundred and seventy-eight (*n* = 378) had complete pre- and postoperative POP-Q data available and were included in the study. The loss of follow-up or missing POP-Q data at 1-year follow-up was 8% in each group. A flow diagram of the study is presented in Fig. 1.

Patient characteristics are compared in Table 1. Patients in the SSH group were older, whereas there was no difference in the body mass index (BMI) or in history of pelvic surgery.

**Table 1:** Patient demographic characteristics

Characteristics	VH (*n* = 196)	SSH (*n* = 182)	*p* value
Age in years	58.1 ± 11.6	63.4 ± 11.3	0.00
BMI in kg/m^2^	25.5 ± 3.8	25.2 ± 3.9	0.39
History of pelvic surgery			
Incontinence surgery	13 (7)	8 (4)	0.38
Colporrhaphy	24 (12)	21 (12)	0.88

Data presented as mean ± standard deviation or number (percentage) as appropriate

*BMI* body mass index, *POP-Q* Pelvic Organ Prolapse Quantification, *VH* vaginal hysterectomy, *SSH* sacrospinous hysteropexy,

*p* value is the level of statistical significance as calculated using the two-sample *t* test for continuous variables and Chi-squared or Fisher’s exact test for categorical variables

Two hundred and eight patients (55%) returned the questionnaires, with no difference in nonresponders between groups. Baseline characteristics of responders were comparable with the complete cohort and nonresponders. In Table 2, questionnaire scores before and 1 year after surgery are shown. The median score on the PGI-I 1 year after surgery showed no difference between VH and SSH, and 77 (73%) and 77 (75%) of patients respectively considered their condition (very) much improved (*p* = 0.86). Four (4%) and six (6%) women receiving VH or SSH respectively considered their condition worse than before the operation. The difference between the groups in UDI, DDI, and IIQ score change between pre- and postoperatively was only statistically significant in favor of VH on the UDI subscale genital prolapse. There were no differences found in the other domains.

**Table 2:** Patient impression of improvement and functional outcome after VH and SSH preoperatively and 1 year postoperatively

Domains	Preoperatively		One year postoperatively		Difference in preoperative and postoperative score			
	VH (*n* = 105)	SSH (*n* = 103)	VH (*n* = 105)	SSH (*n* = 103)		VH	SSH	*p* value
PGI-I			2.0 (1.0–3.0)	2.0 (1.0–2.5)				
PGI-I success			77 (73%)	77 (75%)				
UDI								
Overactive bladder	33 (11–55)	22 (11–44)	22 (0–33)	11 (0–22)	−11(−33 to 0)		−11 (−22 to 0)	0.56
Urinary incontinence	17 (0–50)	17 (0–33)	17 (0–33)	17 (0–17)	0 (−16–0)		0 (−17 to 0)	0.23
Obstructive micturition	17 (0–50)	17 (0–33)	0 (0–33)	0 (0–17)	0 (−33 to 0)		0 (−33 to 0)	0.53
Genital prolapse	67 (33–100)	67 (33–67)	0 (0–8)	0 (0–0)	− 66 (−83 to −33)		−50 (−66 to −17)	0.03
Pain	33 (0–50)	17 (0–33)	0(0–33)	0 (0–17)	−17 (−33 to 0)		0 (−17 to 0)	0.06
DDI								
Constipation	0 (0–17)	0 (0–8)	0 (0–17)	0 (0–0)	0 (−17 to 0)		0 (0 to 0)	0.38
Obstructive defecation	8 (0–17)	0 (0–8)	0 (0–8)	0 (0–8)	0 (−8 to 0)		0 (−6 to 0)	0.50
Pain	0 (0–8)	0 (0–0)	0 (0–0)	0 (0–0)	0 (0 to 0)		0 (0 to 0)	0.06
Incontinence	0 (0–17)	0 (0–0)	0 (0–0)	0 (0–0)	0 (0 to 0)		0 (0 to 0)	0.59
IIQ								
Emotional	22 (0–33)	11 (0–33)	0 (0–22)	0 (0–11)	−11 (−22 to 0)		0 (0 to 0)	0.59
Embarrassement	0 (0–17)	0 (0–17)	0 (0–17)	0 (0–0)	0 (−17 to 0)		0 (−17 to 0)	0.50
Physical	17 (0–33)	0 (0–33)	0 (0–17)	0 (0–0)	0 (−33 to 0)		0 (−17 to 0)	0.17
Mobility	17 (0–33)	11 (0–22)	11 (11–33)	0 (0–11)	−11 (−22 to 0)		−6 (−22 to 0)	0.37
Social	11 (0–22)	0 (0–19)	0 (0–11)	0 (0–0)	0 (−11 to 0)		0 (−11 to 0)	0.89
Sexual functioning								
Sexually active	89 (85)	77 (75)	93 (89)	72 (70)	−4 (%)		5 (%)	0.01
Satisfied with sexual function	3.0 (2.0–3.5)	3.0 (2.0–3.0)	3.0 (2.3–3.0)	3.0 (2.0–3.0)	0 (0.0)		0 (0–0)	0.89
Urine loss during intercourse	21 (20)	13 (13)	17 (16)	5 (5)	−4 (−4)		−8 (8)	0.25
Pain during intercourse	40 (38)	27 (26)	42 (40)	24 (23)	−2 (−2)		3 (3)	0.08

Data presented as numbers (percentage) or median (interquartile range) as appropriate

Scores and percentages were calculated using nonmissing data

*VH* vaginal hysterectomy, *SSH* sacrospinous hysteropexy, *PGI-I* Patient Global Impression of Improvement Index (PGI-I success is defined as a score on PGI-I of 1 or 2, denoting [very] much better), *UDI* Urogenital Distress Inventory, *DDI* Defecation Distress Inventory, *IIQ* Incontinence Impact Questionnaire

*p* value indicates the level of statistical significance calculated using Mann–Whitney *U* test

More women in the VH group became sexually active after surgery than in the SSH group (4 [4%] versus −5 [−5%], *U* = 0.01). Satisfaction with sexual function did not improve after the procedure in either group. Three patients reported de novo dyspareunia after surgery (VH = 2, SSH = 1).

Table 3 shows the POP-Q stages by compartment at baseline and 1 year postoperatively. Most women included had a preoperative POP-Q stage III (i.e., more than 1 cm beyond the hymen). At baseline, there were no differences between the groups in overall POP-Q stage and percentage of patients with vaginal bulge symptoms. However, patients undergoing VH had more concomitant vaginal repairs during surgery than those undergoing SSH (see Table 5).

**Table 3:** Data on anatomical and functional outcome by treatment group at 1 year follow-up

	Preoperatively		One year postoperatively			
	VH (*n* = 196)	SSH (*n* = 182)	*p* value	VH (*n* = 196)	SSH (*p* = 182)	*p* value^a^
Overall POP-Q stage			0.90			< 0.00
0/I	0 (0)	0 (0)		77 (39)	43 (24)	
II	62 (32)	59 (32)		97 (49)	130 (71)	
III	127 (65)	118 (65)		20 (10)	7 (4)	
IV	7 (3)	5 (3)		2 (1)	2 (1)	
POP-Q stage by compartment						
Anterior (Ba ≥ stage 2)	184 (94)	171 (94)	0.96	100 (51)	128 (70)	0.01
Apical (C ≥ stage 2)	138 (70)	92 (51)	0.01	7 (4)	5 (3)	0.64
Posterior (Bp ≥ stage 2)	126 (64)	80 (44)	0.01	37 (19)	30 (16)	0.54
POP beyond the hymen						
Anterior (Ba > 0)	134 (68)	141 (77)	0.05	26 (13)	23 (13)	0.85
Apical (C > 0)	99 (51)	59 (32)	0.01	7 (4)	6 (3)	0.88
Posterior (Bp > 0)	29 (15)	23 (13)	0.54	9 (5)	4 (2)	0.20
Vaginal bulge symptoms	131/150 (91)	114/134 (85)	0.10	28/137 (20)	18/125 (14)	0.20
Repeat POP or UI surgery at 1 year				13 (7)7 (4)	7 (4)	0.69
Composite outcome of surgical success, *N* (%)				126/137 (92)	118/125 (94)	0.44

Data presented as number (percentage)

Vaginal bulge symptoms assessed as positive answer to at least one of the questions “Do you experience a sensation of bulging or protrusion from the vagina?” or “Do you have a bulge or something falling out that you can see in the vagina?”; composite outcome of success was defined as absence of POP beyond the hymen combined with bothersome vaginal bulge symptoms or repeat surgery after treatment

*VH* vaginal hysterectomy, *SSH* sacrospinous hysteropexy, *POP* pelvic organ prolapse, *UI* urinary incontinence

*P* value indicates the test for statistical significance between groups calculated using Chi-squared test

At the 1-year follow-up, there was no difference in the composite outcome of surgical success (126 out of 137 women [92%] after VH, 118 out of 125 women [94%] after SSH; *p* = 0.44). There were no statistically significant differences in the outcomes in the three components of the composite outcome (i.e., POP beyond the hymen, vaginal bulge symptoms postoperatively, repeat surgery for POP).

After SSH, more POP-Q stage 2 or higher was found in the anterior compartment: 128 (70%) compared with 100 (51%) after VH (*p* ≤ 0.05), but this did not lead to a difference in bulge symptoms.

The predictive factors regarding PGI-I score are shown in Table 4. Age, BMI, pre-operative apical prolapse, and the type of surgery were not predictors of a successful outcome.

**Table 4:** Associations of patient characteristics and a positive outcome on the Patient Impression of Improvement, defined as a PGI-I score ≤ 2

Characteristic	Unadjusted OR	*p* value	Adjusted OR	*p* value
Age in years	1.02 [0.98–1.05]	0.31	1.01 [0.98–1.05]	0.46
BMI in kg/m^2^	0.96 [0.88–1.06]	0.41	0.96 [0.87–1.05]	0.36
Apical prolapse preoperative (yes/no)	1.64 [0.80–3.37]	0.18	1.74 [0.80–3.80]	0.16
Type of surgery (VH or SSH)	0.89 [0.44–1.79]	0.74	0.81 [0.37–1.76]	0.59

Apical prolapse is defined as POP-Q point C ≥ −1 cm

*OR* odds ratio, *BMI* body mass index, *VH* vaginal hysterectomy, *SSH* sacrospinous hysteropexy

Table 5 provides details on the intra- and postoperative period. More concomitant repairs were performed in the VH group. Results show less blood loss after SSH and a shorter operating time. The complication rates for VH and SSH were similar, 16 and 17% respectively. The most frequent complication was temporary urinary retention treated with a transurethral catheter or clean intermittent self-catheterization. Five serious adverse events (Clavien–Dindo grade 3 or 4) were reported: laparotomy for ongoing pain and abscess formation (VH), laparotomy for mechanical ileus (VH), admission to the intensive care unit because of postoperative cardiovascular complications (SSH), re-admission because of a pulmonary embolism (SSH), and an episode of atrial fibrillation after surgery (SSH).

**Table 5:** Details on intra- and postoperative period

Characteristics	VH (*n* = 196)	SSH (*n* = 182)	*p* value
Concomitant repair			
Anterior colporrhaphy	48 (24)	115 (63)	0.00*
Anterior and posterior	109 (56)	26 (14)	0.00*
Posterior colporrhaphy	11 (6)	11 (6)	0.98*
Blood loss in ml	173 ± 140	88 ± 178	< 0.00**
Operating time in min	90 ± 37	59 ± 23	< 0.00**
Length of hospital stay in days	3.3 ± 1.8	2.4 ± 1.8	< 0.00**
Re-admission	6 (3)	5 (3)	0.76*
Patients with at least one complication	31 (16)	31 (17)	0.98***
Clavien–Dindo classification			0.39***
Grade 1	19 (10)	23 (13)	
Grade 2	10 (5)	5 (3)	
Grade 3	1 (1)	3 (2)	
Grade 4	1 (1)	0 (0)	
Grade 5	0 (0)	0 (0)	
Complications by category ^a^			
Urological (retention or urinary tract infection)	25	21	
Chronic pelvic pain	0	1	
Infection	1	2	
Post-operative hemorrhage or hematoma	2	4	
Cardiovascular	0	2	
Gastro-intestinal	1	0	
Other			
Pulmonary embolism	0	1	
Tampon accidentally in situ > 2 days	0	2	
Repeat surgery to release stitches	NA	1	
Temporary neurological deficit of one leg	0	1	
Perforation			
Rectal serosa	1	0	
Bladder	0	1	

Clavien–Dindo classification of surgical complications as described by Clavien et al. [16]

Data presented as mean ± standard deviation or number (percentage) as appropriate

*VH* vaginal hysterectomy, *SSH* sacrospinous hysteropexy, *MUS* midurethral sling, *NA* not applicable

*Calculated using Fisher's exact test

**Calculated using independent sample *t* test

***Calculated using Chi-squared test

^a^Patients can appear in multiple categories

Table 6 provides details regarding repeat surgery within 1 year of surgery. After VH, 8 patients underwent native tissue repair or vaginal mesh surgery in a previous operated compartment, compared with 3 patients after SSH.

**Table 6:** Details on patients undergoing repeat surgery within 1 year after surgery

	VH, *n* = 13 (7%)	SSH, *n* = 7 (4%)
Midurethral sling	0	1 (14)
Vaginal hysterectomy	NA	2 (29)
Native tissue repair	4 (31)	3 (43)
Anterior repair	2 (treated compartment 2)	2 (treated compartment 2)
Posterior repair	1 (treated comp. 1)	1 (treated compartment 0)
Anterior and posterior repair	1 (treated compartment 1)	0
Vaginal mesh surgery	7 (54)	2 (29)
Anterior	1 (treated compartment 1)	2 (treated compartment 1)
Posterior	3 (treated compartment 1)	0
Total mesh	3 (treated compartment 2)	0
Laparoscopic sacropexy	2 (15)	1 (14)

Data presented as number (percentage)

*VH* vaginal hysterectomy, *SSH* sacrospinous hysteropexy, *NA* not applicable

## Discussion

Our study shows that the type of surgery, VH or SSH, exerted no effect on patient impression of improvement at 1 year postoperatively. Three quarters of women felt that their condition had (very) much improved. There was also no difference in the composite outcome of surgical success, defined absence of POP beyond the hymen in combination with bothersome bulge symptoms, or repeat surgery within 1 year. Over 90% in both groups reached this definition of success. However, more colporrhaphies were needed in the VH group to reach this outcome, and VH took 30 min longer to perform.

As uterine-preserving surgical techniques for uterovaginal POP repair gain popularity, patients need new information on the advantages and disadvantages of each procedure. In our opinion, adding the PGI-I to the questionnaire is a valuable addition, because it is a simple and easy scale that measures general impression of improvement in one question. Although it does not obtain detailed information, it is intuitively understandable to clinicians and patients [15]. Our results regarding functional outcome after surgery are in line with those in the existing literature. Both VH and SSH show a significant improvement in all domain scores and thus improve quality of life in patients [4–6]. There was a slightly better outcome after VH in one domain, i.e., UDI genital prolapse, and in the number of women being sexually active. The scores on sexual functioning did not improve in either group after the procedure. More research is needed to identify reasons and to propose solutions to this problem.

Previous studies show conflicting results regarding anatomical outcome. Detollenaere et al. [7] found that sacrospinous hysteropexy was non-inferior to vaginal hysterectomy concerning surgical failure of the apical compartment, whereas Dietz et al. [9] concluded that SSH led to a higher recurrence rate of apical prolapse than VH 12 months after surgery. The low effectiveness of SSH in the study by Dietz et al. [9]has not been fully unraveled. Differences are most likely related to different ranges for objective success rates, which makes comparison between different methods in the literature difficult. We observed that patients with a baseline POP-Q stage ≥ 2 but without apical prolapse also regularly received VH or SSH. This is why in our study, unlike Detollenaere et al. [7] and Dietz et al. [9], we included all patients with a POP-Q stage ≥ 2 in at least one compartment. Barber et al. stated that the absence of vaginal bulge symptoms postoperatively has a significant relationship with a patient's assessment of overall improvement, whereas anatomical success alone does not [11]. Therefore, we defined POP recurrence as an overall POP stage ≥ 2 combined with bothersome vaginal bulge symptoms or repeat surgery. We found a POP recurrence rate of 3% and 4% after respectively VH and SSH. These results are in line with the conclusion of Detollenaere et al. [7], which confirms the hypothesis that SSH might be non-inferior to VH with regard to clinically relevant recurrence. Our results concerning postoperative complications are also in line with those of the previous literature [19].

During our study period, the Manchester procedure (MP) uterine-preserving technique was gaining popularity, and a large RCT on MP versus SSH has recently been published [8]. That study showed the superiority of MP in terms of a composite outcome. Only a little scientific evidence on the comparison between MP and VH is available. In a recent study by Tolstrup et al. [8], MP was also superior to VH. This conclusion was, however, only based on anatomical recurrence. In a cohort study, Enklaar et al. [20] found no significant difference in subjective recurrence between the modified MP and VH, defined as recurrence of bulge symptoms. In a patient preference study, Schulten et al. [21] concluded that the preference of women for MP or SSH was almost equally divided after counseling. A secondary analysis of the available studies combined might identify subgroups that would benefit most from a VH, SSH or MP. Such evidence is needed to implement individualized health care.

We found that peri-operative outcomes, such as blood loss, operating time, and hospital stay, were all in favor of SSH. However, in particular, the difference in blood loss of less than 100 ml is not expected to be clinically relevant. The data on (difference in) hospital stay from this study seem outdated, as the average hospital stay is much shorter nowadays (i.e., 1 day).

Strengths of this study are the large sample collected over a long period, and the multicenter design. Furthermore, by adding the PGI-I and using a relevant composite outcome to assess POP recurrence, we made outcomes clinically relevant and comparable with those of other studies. We expect a low risk of response bias as the responders were similar to nonresponders. A previous study has reported a broad range of responses rates, from 6 to 70% [22]; thus, this study is situated at the higher end. Loss to follow up regarding the physical examination was identical in the groups (8%). A limitation of the study is the retrospective design, irrespective of the prospectively collected data. Moreover, this study has a relatively short follow-up period of 1 year. A limitation is that in this study, choice of procedure was based on surgeon and patient preference, as there were no criteria leading to a choice. During the first half of our study period, more VHs were performed, but this slowly shifted toward more SSHs. There must have been a change in counseling by gynecologists.

In conclusion, patient impression of improvement and functional outcome were similar 1 year after SSH and VH. The decision on the surgical approach should therefore not be based on a foreseeable difference in patient impression of improvement or composite outcome of surgical success. Other factors may, however, play a role, such as the patient’s wish to remove or preserve the uterus (e.g., in the case of menorrhagia). The results of this study will help patients and their gynecologists to make a well-considered choice of surgical procedure.

## Data Availability

The data that support the findings of this study are available from the corresponding author, L.M. Stoter, upon reasonable request.
